# Supplementary material for: Association between oral microbiome and seven types of cancers in East Asian population: a two-sample Mendelian randomization analysis
Source: Front Mol Biosci. 2023 Nov 21;10:1327893. doi: 10.3389/fmolb.2023.1327893 (PMC10702768; doi:10.3389/fmolb.2023.1327893)
Supplement: Supplementary file 1 [file DataSheet1.ZIP › Supplementary Materials/MR plots of saliva/saliva═╝/Lung Cancer/pheno.2776_to_lung cancer_forest.pdf]

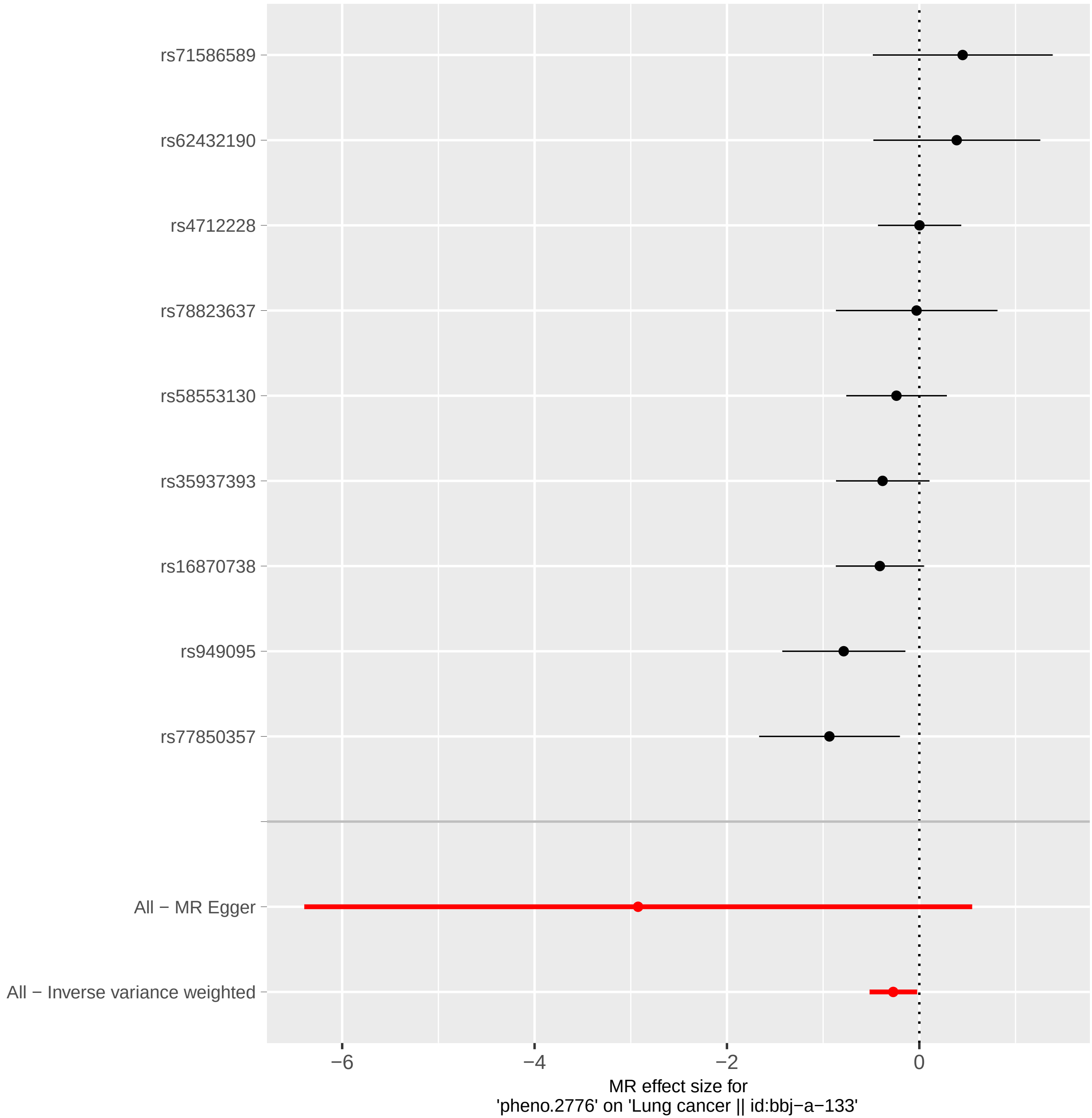
rs71586589
rs62432190
rs4712228
rs78823637
rs58553130
rs35937393
rs16870738
rs949095
rs77850357
All − MR Egger
All − Inverse variance weighted
−6
−4
−2
0
MR effect size for
'pheno.2776' on 'Lung cancer || id:bbj−a−133'
